# Evaluation of inhaled nitric oxide (iNO) treatment for moderate-to-severe ARDS in critically ill patients with COVID-19: a multicenter cohort study

**DOI:** 10.1186/s13054-022-04158-y

**Published:** 2022-10-03

**Authors:** Khalid Al Sulaiman, Ghazwa B. Korayem, Ali F. Altebainawi, Shmeylan Al Harbi, Abdulrahman Alissa, Abdullah Alharthi, Raed Kensara, Amjaad Alfahed, Ramesh Vishwakarma, Hussain Al Haji, Naif Almohaimid, Omar Al Zumai, Fahad Alrubayan, Abdulmajid Asiri, Nasser Alkahtani, Abdulaziz Alolayan, Samiah Alsohimi, Nawal Melibari, Alaa Almagthali, Seba Aljahdali, Abeer A. Alenazi, Alawi S. Alsaeedi, Ghassan Al Ghamdi, Omar Al Faris, Joud Alqahtani, Jalal Al Qahtani, Khalid A. Alshammari, Khalil I. Alshammari, Ohoud Aljuhani

**Affiliations:** 1 Pharmaceutical Care Department, King Abdulaziz Medical City, Riyadh, Saudi Arabia; 2 College of Pharmacy, King Saud Bin Abdulaziz University for Health Sciences, Riyadh, Saudi Arabia; 3 King Abdullah International Medical Research Center, King Abdulaziz Medical City (KAMC), Ministry of National Guard Health Affairs, Riyadh, Saudi Arabia; 4 Department of Pharmacy Practice, College of Pharmacy, Princess Nourah Bint Abdulrahman University, P.O.Box 84428, Riyadh, 11671 Saudi Arabia; 5 Pharmaceutical Care Services, King Salman Specialist Hospital, Hail Health Cluster, Hail, Saudi Arabia; 6 Pharmaceutical Care Services, King Abdullah bin Abdulaziz University Hospital, Riyadh, Saudi Arabia; 7 Pharmaceutical Care Department, King Abdulaziz Medical City, Jeddah, Saudi Arabia; 8 Statistics Department, European Organization for Research and Treatment of Cancer (EORTC) Headquarters, Brussels, Belgium; 9 Respiratory Department, King Abdulaziz Medical City, Riyadh, Saudi Arabia; 10 Intensive Care Department, King Abdulaziz Medical City, Riyadh, Saudi Arabia; 11 College of Medicine, King Saud Bin Abdulaziz University for Health Sciences, Riyadh, Saudi Arabia; 12 Pharmaceutical Services Department, King Abdulaziz University Hospital, Jeddah, Saudi Arabia; 13 Pharmaceutical Care Department, Prince Sultan Military Medical City, Riyadh, Saudi Arabia; 14 Department of Internal Medicine, Al Imam Mohammad Ibn Saud Islamic University (IMSIU), Riyadh, Saudi Arabia; 15 Respiratory Department, King Abdulaziz Medical City, Jeddah, Saudi Arabia; 16 Department of Pharmacy Practice, Faculty of Pharmacy, King Abdulaziz University, Jeddah, Saudi Arabia; 17 Phamacy Department, King Fahad Armed Forces Hospital, Jeddah, Saudi Arabia; 18 Saudi Critical Care Pharmacy Research (SCAPE) Platform, Riyadh, Saudi Arabia

**Keywords:** COVID-19, SARS-CoV-2, Acute respiratory distress syndrome (ARDS), Inhaled nitric oxide, iNO, Oxygenation parameter, Critically ill, Intensive care units (ICUs), 30-day mortality, In-hospital mortality

## Abstract

**Background:**

Inhaled nitric oxide (iNO) is used as rescue therapy in patients with refractory hypoxemia due to severe COVID-19 acute respiratory distress syndrome (ARDS) despite the recommendation against the use of this treatment. To date, the effect of iNO on the clinical outcomes of critically ill COVID-19 patients with moderate-to-severe ARDS remains arguable. Therefore, this study aimed to evaluate the use of iNO in critically ill COVID-19 patients with moderate-to-severe ARDS.

**Methods:**

This multicenter, retrospective cohort study included critically ill adult patients with confirmed COVID-19 treated from March 01, 2020, until July 31, 2021. Eligible patients with moderate-to-severe ARDS were subsequently categorized into two groups based on inhaled nitric oxide (iNO) use throughout their ICU stay. The primary endpoint was the improvement in oxygenation parameters 24 h after iNO use. Other outcomes were considered secondary. Propensity score matching (1:2) was used based on the predefined criteria.

**Results:**

A total of 1598 patients were screened, and 815 were included based on the eligibility criteria. Among them, 210 patients were matched based on predefined criteria. Oxygenation parameters (PaO_2_, FiO_2_ requirement, P/F ratio, oxygenation index) were significantly improved 24 h after iNO administration within a median of six days of ICU admission. However, the risk of 30-day and in-hospital mortality were found to be similar between the two groups (HR: 1.18; 95% CI: 0.77, 1.82; *p* = 0.45 and HR: 1.40; 95% CI: 0.94, 2.11; *p*= 0.10, respectively). On the other hand, ventilator-free days (VFDs) were significantly fewer, and  ICU and hospital LOS were significantly longer in the iNO group. In addition, patients who received iNO had higher odds of acute kidney injury (AKI) (OR (95% CI): 2.35 (1.30, 4.26), *p* value = 0.005) and hospital/ventilator-acquired pneumonia (OR (95% CI): 3.2 (1.76, 5.83), *p* value = 0.001).

**Conclusion:**

In critically ill COVID-19 patients with moderate-to-severe ARDS, iNO rescue therapy is associated with improved oxygenation parameters but no mortality benefits. Moreover, iNO use is associated with higher odds of AKI, pneumonia, longer LOS, and fewer VFDs.

**Supplementary Information:**

The online version contains supplementary material available at 10.1186/s13054-022-04158-y.

## Introduction

One of the major complications of coronavirus disease (COVID-19) is acute hypoxemic respiratory failure requiring mechanical ventilation [1, 2]. This complication can increase mortality rates, particularly in patients who require mechanical ventilation [2–5]. It is estimated that the mortality rate in patients with COVID-19 and acute respiratory distress syndrome (ARDS) ranges between 26 and 88.3% [6–9]. ARDS involves an acute alveolar-capillary membrane inflammatory response that is characterized by poor oxygenation and pulmonary infiltrates, resulting in "stiffness" of the lungs leading to hypoxic failure [10, 11]. ARDS can cause an imbalance between ventilation and perfusion, resulting in intensified intrapulmonary shunting in nonventilated lung regions from pulmonary vasodilation and vasoconstriction in ventilated zones, as well as pulmonary hypertension [12].

There is no specific pharmacologic agent that treats ARDS, and the management mainly consists of supportive lung-protective ventilation to minimize lung injury [11]. Multiple rescue therapies, including neuromuscular blockade and inhaled pulmonary vasodilators such as inhaled nitric oxide (iNO), have been used in refractory hypoxemia ARDS [13]. Inhaled nitric oxide may improve ventilation and overcome perfusion imbalance and pulmonary vascular resistance, relieving hypoxemia caused by ARDS [14]. A previous meta-analysis including 14 studies reported that iNO was not associated with any mortality benefit in patients with ARDS [15]. Nonetheless, it had a transient positive effect on oxygenation, suggesting that there is a potential benefit of iNO in this patient population [15].

The evidence regarding the safety and efficacy of iNO in patients with ARDS due to COVID-19 is limited and contradictory [16]. In two observational studies, the use of iNO led to significant improvement in oxygenation and the PaO_2_/FiO_2_ ratio [17, 18]. In contrast, Tavazzi et al. did not find any significant benefit of oxygenation in patients with COVID-19 and refractory hypoxemia who received iNO as rescue therapy [19]. A subanalysis of a systematic review of inhaled pulmonary vasodilators, including four trials of iNO, did not find any improvement in oxygenation with the use of iNO as rescue therapy among patients with COVID-19 and refractory hypoxemia [20]. Therefore, several guidelines recommend against the routine use of iNO in mechanically ventilated patients with COVID-19 ARDS [21, 22]. However, in mechanically ventilated patients with refractory hypoxemia due to severe COVID-19 ARDS, iNO may be used as a rescue therapy.

Despite the limited evidence regarding the benefit of iNO, clinicians continue to use it as a last resort for mechanically ventilated patients with ARDS and COVID-19. To date, the effect of iNO on the clinical outcomes of critically ill patients with COVID-19 and moderate-to-severe ARDS remains arguable. Thus, the objective of this study was to evaluate the use of iNO in critically ill COVID-19 patients with moderate-to-severe ARDS.

## Methods

### Study design

A multicenter, retrospective cohort study all critically ill adult patients with confirmed COVID-19 who were admitted to intensive care units (ICUs) from March 01, 2020, until July 31, 2021. The patients were diagnosed with COVID-19 using reverse transcriptase-polymerase chain reaction (RT‒PCR) nasopharyngeal or throat swabs. The eligible patients were categorized into two groups based on iNO use during the ICU stay (control vs. iNO). All patients were followed until they were discharged from the hospital or died during their stay. The study was approved by King Abdullah International Medical Research Center (KAIMRC) in December 2020 (Ref.# RC20. 638.R). The IRB committee waived informed consent from the study patients due to the retrospective observational nature of the study. All methods were performed in accordance with relevant guidelines and regulations.

### Study participants

**Fig. 1:**
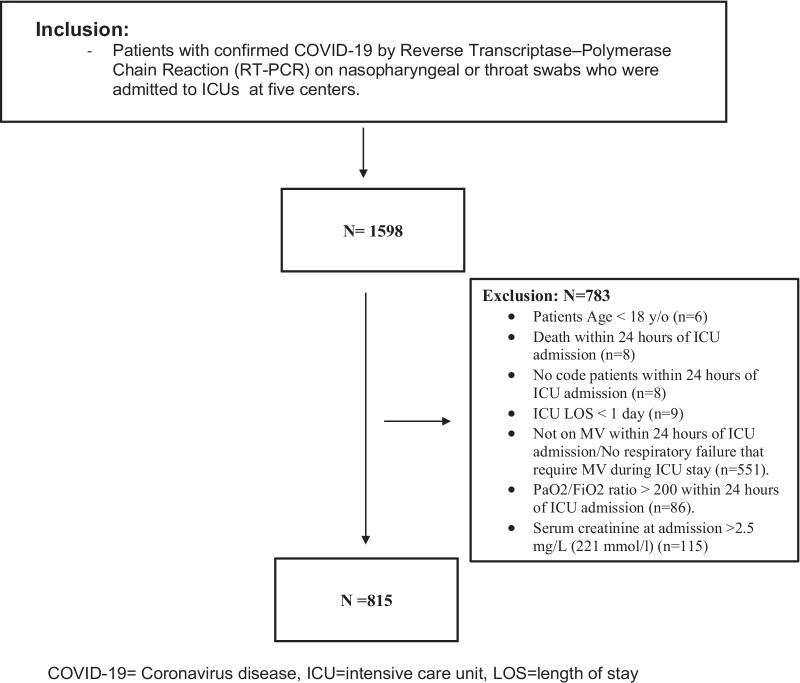
Flow diagram showing patients recruited with COVID-19

We included adult patients (age ≥ 18 years) admitted to the ICUs with confirmed COVID-19. Patients were excluded if they did not develop respiratory failure that required mechanical ventilation (MV) during the ICU stay, were not on MV at admission, had a PaO_2_/FiO_2_ ratio > 200 within 24 h of ICU admission, had serum creatinine > 2.5 mg/dL (221 mmol/l), had an ICU length of stay (LOS) ≤ one day, died within the first 24 h of ICU admission or were labeled "Do-Not-Resuscitate" (Fig. 1).

### Study setting

The study took place at five hospitals in Saudi Arabia: King Abdulaziz Medical City (Riyadh & Jeddah), King Abdulaziz University Hospital (Jeddah), King Abdullah bin Abdulaziz University Hospital (KAAUH) (Riyadh), and King Salman Specialist Hospital (Hail). These centers were selected based on the geographic distribution, the availability of electronic records, and the center's willingness to participate in the national project. The primary site for this multicenter retrospective study was King Abdulaziz Medical City (Riyadh), a tertiary care center.

### Inhaled nitric oxide administration

Nitric oxide emanates in gas form and is stored in cylinders. The gas regulator should be attached to the nitric oxide (NO) cylinder, and the cylinder should have sufficient pressure to initiate and maintain the therapy throughout the procedure. Calibration of the O_2_ analyzer is essential before connecting the mechanical ventilator inspiratory limb (to the patient) and then the NO and NO2 analyzer afterward. In this study, the gas sampling line was attached to the back of the analyzer, and then the gas sampling line was attached to the ventilator inspiratory limb just before the humidifier. The initial dose of iNO that is institutionally applied in clinical practice is 20 parts per million (PPM) following AARC clinical guidelines in severe ARDS adult patients, whereas 40 PPM is the maximum dose. However, observation of patient response to any complications related to iNO use and avoiding overdose is needed. Excess doses of iNO may cause methemoglobinemia (MetHb) and result in a drop in blood pressure [23–25].

### Data collection

Each participant's data were collected and controlled using King Abdullah International Medical Research Center's Research Electronic Data Capture (REDCap®) software. We collected patients' demographic data, comorbidities, vital signs and laboratory tests, baseline severity scores (i.e., Acute Physiology and Chronic Health Evaluation II (APACHE II), Sequential Organ Failure Assessment (SOFA), Nutrition Risk in the Critically Ill (*NUTRIC*) and multiple organ dysfunction scores), Glasgow Coma Score (GCS), acute kidney injury status, use of prone positioning, receipt of mechanical ventilation (MV), MV parameters (e.g., PaO_2_/FiO_2_ ratio, FiO_2_ requirement) and oxygenation index (OI) within 24 h of ICU admission. Moreover, renal profile, liver function tests (LFTs), coagulation profile (i.e., INR, aPTT, fibrinogen, D-dimer), and inflammatory markers (ferritin, procalcitonin, and creatine phosphokinase (CPK)) within 24 h of ICU admission were collected. In addition, oxygenation parameters (i.e., PaO_2_, PaCO_2_, oxygenation index (OI), PaO_2_/FiO_2_ ratio, and FiO_2_ requirement) were collected pre- and 24 h post-iNO use. Last, the use of tocilizumab, corticosteroids, and inhaled nitric oxide (timing to initiation, dose, and duration) during the ICU stay was recorded for the eligible patients.

### Outcomes

The primary endpoint was the improvement in oxygenation parameters 24 h after iNO use. The secondary endpoints were 30-day and in-hospital mortality, hospital LOS, ICU LOS, number of ventilator-free days (VFDs) at 30 days, and ICU-acquired complication**s** (new-onset atrial fibrillation, thrombosis, acute kidney injury (AKI), liver injury, hospital/ventilator-acquired pneumonia, and secondary fungal infection). (Additional file 1) [26–28].

### Statistical analysis

Continuous variables are presented as the mean with standard deviation (SD) or the median with lower and upper quartiles (Q1, Q3), and categorical variables are presented as a number with a percentage as appropriate. All numerical variables' normality assumptions were assessed using a statistical test (i.e., Shapiro–Wilk test) and graphical representation (i.e., histograms and Q–Q plots). In addition, model fit was assessed using the Hosmer‒Lemeshow goodness-of-fit test.

Baseline and outcome variables were compared between the two study groups. The Chi-square or Fisher exact test was used for categorical variables. Normally distributed continuous variables were compared using Student’s *t* test, and the Mann‒Whitney *U* test was used to compare non-normally distributed variables. Multivariable Cox proportional hazards regression analyses were performed for 30-day and in-hospital mortality. The proportionality assumption was assessed before fitting the Cox model. Visual assessment was performed to assess the assumption by plotting log(-log) plots and testing the correlation of scaled Schoenfeld residuals with rank-ordered time. Multivariable logistic regression and negative binomial regression analysis were used for the other outcomes considered in this study as appropriate. The odds ratios (OR) or estimates with the 95% confidence intervals (CI) were reported as appropriate. No imputation was made for missing data, as the cohort of patients in our study was not derived from random selection.

The propensity score matching procedure (Proc PS match) (SAS, Cary, NC) was used to match patients (1:2 ratio) who received inhaled nitric oxide therapy (active group) to patients who did not (control group). Regression analysis was performed for the study outcomes after considering PS scores as covariates in the model. These PS scores were generated through propensity score analysis after considering all relevant covariates, which included the patient's age, BMI, early use of dexamethasone and tocilizumab within 24 h of ICU admission, SOFA score, serum creatinine, PaO_2_/FiO_2_ ratio at admission, pulmonary hypertension, and right heart failure [29–31]. Patients were matched only if the difference in the logits of the propensity scores for pairs of patients from the two groups was less than or equal to 0.1 times the pooled estimate of the standard deviation. A greedy nearest neighbor matching method was used in which one patient who received inhaled nitric oxide (active) was matched with two patients who did not (control), which eventually produced the smallest within-pair difference among all available pairs with treated patients.

## Results

During the study period, 1598 patients were screened. Based on the eligibility criteria, we included a total of 815 patients in our analysis (Fig. 1). Among them, 76 (9.3%) received inhaled nitric oxide (iNO), and of these, twenty-seven patients were considered iNO responders. After propensity score matching (1:2 ratio), we included 210 patients based on predefined criteria. Patients were given iNO with a median dose of 40.0 (32.5, 40.0) parts per million (PPM) for a median duration of 85.0 (37.0, 148.0) hours. The median time for iNO initiation from ICU admission was six days.

### Demographic and clinical characteristics

Most patients in both arms (60.2%) were male, with a mean age of 62.5 (SD ± 14.3). Diabetes mellitus (DM) (58.2%), hypertension (HTN) (55.5%), and dyslipidemia (22%) were the most prevalent underlying comorbidities in our patients. Before propensity score (PS) matching, there was a notable difference between the groups, which demonstrated that patients in the iNO group received more inotrope support, nephrotoxic drugs/material, pharmacologic deep vein thrombosis (DVT) prophylaxis, and early use of tocilizumab and dexamethasone therapy. In addition, the patients who received iNO had a higher body mass index (BMI), and pulmonary HTN. Of interest, those who received iNO had more DVT and liver disease as a comorbid condition before PS matching. After PS matching, most of the patients' characteristics were found to be comparable between the two groups, except that patients who received iNO had a lower baseline C-reactive protein (CRP) and higher liver disease as comorbid conditions (Table 1).

**Table 1:** Summary of demography and baseline characteristics

	Before propensity score (PS)				After propensity score (PS)			
	Overall (*N* = 815)	Control (*N* = 739)	iNO (*N* = 76)	*p*-value	Overall (210)	Control (*N* = 140)	iNO (*N* = 70)	*p*-value
Age (years), mean (SD)	62.5 (14.27)	62.7 (14.33)	61.0 (13.69)	0.2474^	60.1 (14.40)	60.2 (14.93)	60.0 (13.38)	0.9327*
Gender – male, *n* (%)	488 (60.2)	447 (60.7)	41 (54.7)	0.3066^^	120 (57.1)	80 (57.1)	40 (57.1)	> 0.9999^^
Weight (kg), mean (SD)	82.0 (19.04)	81.4 (18.05)	88.2 (26.28)	0.0604^	87.2 (22.86)	86.0 (20.92)	89.7 (26.41)	0.5159^
Body mass index (BMI), mean (SD)	31.5 (9.02)	31.3 (8.88)	33.7 (10.03)	0.0427^	33.6 (9.20)	33.5 (8.65)	33.9 (10.26)	0.8059^
APACHE II score, median (Q1,Q3)	15.0 (10.00, 22.00)	15.0 (10.00, 22.00)	15.0 (9.50, 21.00)	0.8583^	14.0 (10.00, 21.00)	14.0 (10.00, 21.00)	15.0 (9.00, 21.00)	0.9681^
SOFA score, median (Q1,Q3)	5.0 (3.00, 7.00)	5.0 (3.00, 7.00)	5.0 (4.00, 7.50)	0.2367^	5.0 (4.00, 8.00)	5.0 (4.00, 8.00)	5.0 (4.00, 8.00)	0.6282^
Multiple organ dysfunction (MOD) score at admission, median (Q1,Q3)	5.0 (4.00, 7.00)	5.0 (4.00, 7.00)	6.0 (5.00, 8.00)	0.3732^	6.0 (5.00, 8.00)	6.0 (5.00, 7.00)	6.0 (5.00, 8.50)	0.8699^
Early use of dexamethasone within 24 h, n (%)	539 (66.1)	482 (65.2)	57 (75.0)	0.0864^^	156 (74.3)	102 (72.9)	54 (77.1)	0.5029^^
Early use of tocilizumab within 24 h, n (%)	174 (21.3)	150 (20.3)	24 (31.6)	0.0223^^	76 (36.2)	54 (38.6)	22 (31.4)	0.3099^^
Serum creatinine (mmol/L) at admission, median (Q1,Q3)	86.0 (67.00, 116.00)	86.0 (67.00, 115.00)	87.5 (67.00, 127.50)	0.3247^	87.5 (67.00, 134.00)	87.0 (66.50, 134.00)	91.5 (68.00, 133.00)	0.6028^
Blood urea nitrogen (BUN) at admission (mmol/l), median (Q1,Q3)	6.8 (4.80, 10.00)	6.8 (4.80, 10.10)	6.0 (4.80, 9.30)	0.2394^	6.5 (4.70, 10.30)	6.8 (4.35, 10.70)	6.0 (4.90, 9.30)	0.3616^
Acute Kidney Injury (AKI) within 24 h of ICU admission, n (%)	229 (28.3)	204 (27.8)	25 (32.9)	0.3509^^	64 (30.6)	40 (28.8)	24 (34.3)	0.4148^^
PaO_2_/FiO_2_ ratio within 24 h of admission, Median (Q1,Q3)	77.6 (59.30, 109.17)	77.7 (59.25, 110.00)	74.4 (59.82, 98.00)	0.4691^	71.1 (58.00, 99.71)	70.7 (57.50, 101.75)	72.9 (59.33, 95.29)	0.9443^
A-A gradient, median (Q1,Q3)	456.2 (327.06, 579.88)	450.7 (325.57, 579.65)	487.7 (369.68, 587.59)	0.1639^	474.2 (337.09, 589.60)	461.7 (324.66, 585.20)	492.1 (395.67, 600.70)	0.2523^
Oxygenation index (OI), median (Q1,Q3)	19.0 (12.16, 28.72)	18.8 (12.15, 28.41)	22.9 (12.73, 32.63)	0.4719^	22.4 (12.73, 29.51)	20.5 (12.54, 28.91)	23.1 (12.73, 37.04)	0.5806^
Inotropes/vasopressors use within 24 h of admission, *n* (%)	228 (28.3)	200 (27.4)	28 (36.8)	0.0806^^	69 (33.0)	45 (32.4)	24 (34.3)	0.7815^^
Lactic acid baseline (mmol/l), median (Q1,Q3)	1.8 (1.31, 2.50)	1.7 (1.32, 2.46)	1.8 (1.23, 2.75)	0.4958^	2.0 (1.39, 2.67)	2.0 (1.40, 2.60)	1.9 (1.30, 2.75)	0.7388^
Platelets count baseline (10^9/l), median (Q1,Q3)	242.0 (187.00, 316.00)	244.0 (187.00, 318.00)	232.0 (178.00, 292.00)	0.3632^	238.0 (182.00, 319.50)	241.0 (184.00, 331.00)	228.0 (175.00, 280.00)	0.1989^
Total WBC baseline (10^9/l), median (Q1,Q3)	9.6 (6.47, 12.80)	9.7 (6.59, 12.90)	9.0 (5.40, 12.05)	0.1128^	9.1 (5.80, 12.60)	9.3 (6.31, 12.80)	8.7 (5.22, 11.80)	0.1602^
International normalized ratio (INR), median (Q1,Q3)	1.1 (1.00, 1.20)	1.1 (1.01, 1.20)	1.0 (0.99, 1.12)	0.0050^	1.1 (1.00, 1.14)	1.1 (1.00, 1.15)	1.0 (0.98, 1.12)	0.1956^
Activated partial thromboplastin time (aPTT) baseline (Seconds), median (Q1,Q3)	30.0 (26.70, 33.90)	30.1 (26.70, 33.90)	29.4 (26.00, 33.80)	0.4558^	29.5 (26.35, 32.90)	29.3 (26.60, 32.60)	29.6 (26.00, 33.80)	0.6378^
Total bilirubin (umol/l), median (Q1,Q3)	9.2 (6.70, 13.70)	9.2 (6.70, 13.60)	9.6 (6.90, 15.00)	0.2665^	8.9 (6.80, 14.80)	8.8 (6.65, 14.65)	9.5 (7.00, 15.00)	0.3082^
Alanine transaminase (ALT) at admission (U/L), median (Q1,Q3)	37.0 (25.00, 58.00)	37.0 (24.00, 57.00)	38.0 (25.00, 62.00)	0.7194^	35.0 (24.00, 60.00)	34.5 (23.00, 53.00)	38.0 (28.00, 62.00)	0.1851^
Aspartate transaminase (AST) at admission (U/L), median (Q1,Q3)	51.0 (34.00, 75.50)	51.0 (34.00, 74.00)	56.0 (36.00, 88.00)	0.2071^	52.0 (35.00, 77.00)	50.0 (34.00, 72.00)	57.0 (40.00, 90.00)	0.0793^
Troponin I, median (Q1,Q3)	32.3 (15.00, 99.05)	30.0 (14.80, 98.10)	55.1 (22.00, 113.70)	0.1550^	46.4 (20.40, 148.20)	31.4 (14.76, 151.00)	55.8 (26.40, 113.70)	0.1916^
Albumin baseline (gm/l), median (Q1,Q3)	33.0 (29.00, 36.00)	33.0 (29.00, 36.00)	33.0 (31.00, 35.00)	0.8017^	33.0 (30.00, 35.00)	32.5 (30.00, 35.00)	33.0 (31.00, 35.00)	0.3480*
Hematocrit at admission (L/L), median (Q1,Q3)	0.4 (0.35, 0.43)	0.4 (0.35, 0.43)	0.4 (0.35, 0.43)	0.8675^	0.4 (0.35, 0.43)	0.4 (0.36, 0.43)	0.4 (0.35, 0.43)	0.9211*
Creatine phosphokinase (CPK) baseline (U/l), median (Q1,Q3)	159.5 (65.00, 401.50)	151.0 (63.00, 402.00)	197.0 (74.00, 398.00)	0.2345^	197.0 (74.00, 477.00)	174.5 (63.00, 494.00)	215.0 (102.00, 398.00)	0.5771^
C-reactive protein (CRP) baseline (mg/l), median (Q1,Q3)	131.0 (73.80, 202.00)	133.0 (74.00, 203.00)	119.5 (66.50, 174.50)	0.3260^	148.0 (83.00, 256.00)	161.0 (94.00, 275.00)	121.0 (67.00, 179.00)	0.0093^
ESR at admission (mm/hr), median (Q1,Q3)	77.5 (51.00, 99.00)	79.0 (51.00, 99.00)	68.0 (50.00, 105.00)	0.5353^	71.0 (49.50, 90.50)	75.5 (49.00, 92.00)	66.5 (50.00, 89.00)	0.7781*
Procalcitonin (ng/ml), median (Q1,Q3)	0.4 (0.15, 1.12)	0.4 (0.14, 1.14)	0.3 (0.19, 1.09)	0.4899^	0.4 (0.18, 1.37)	0.6 (0.14, 1.39)	0.3 (0.19, 1.09)	0.9696^
Fibrinogen level baseline (gm/l), median (Q1,Q3)	5.4 (3.96, 7.00)	5.5 (3.89, 7.02)	5.0 (4.21, 6.46)	0.5645^	5.4 (3.91, 6.91)	5.9 (3.73, 7.02)	4.8 (4.08, 6.46)	0.3245^
D-dimer level baseline (mg/l), median (Q1,Q3)	1.2 (0.68, 3.10)	1.3 (0.69, 3.10)	1.1 (0.58, 2.48)	0.2365^	1.4 (0.70, 3.29)	1.5 (0.77, 3.48)	1.1 (0.58, 3.27)	0.1428^
Ferritin level baseline (ug/l), median (Q1,Q3)	658.4 (338.15, 1480.40)	651.0 (335.30, 1448.40)	878.6 (400.00, 2179.30)	0.1683^	780.6 (359.00, 2106.00)	757.0 (352.80, 1763.00)	966.3 (400.00, 2241.50)	0.5919^
Blood glucose level baseline (mmol/l), median (Q1,Q3)	11.3 (7.90, 16.20)	11.1 (7.85, 16.10)	12.5 (8.00, 17.30)	0.2740^	12.3 (8.50, 17.25)	12.2 (8.50, 17.20)	12.5 (8.40, 17.30)	0.9007^
Respiratory rate (breath per minute) at admission, median (Q1,Q3)	28.0 (24.00, 34.00)	28.0 (24.00, 34.00)	30.0 (25.00, 35.00)	0.1598^	30.0 (25.00, 35.00)	30.0 (25.50, 36.00)	30.0 (25.00, 35.00)	0.5234*
Highest heart rate (HR) at admission (BPM), median (Q1,Q3)	103.0 (91.00, 115.00)	103.0 (91.00, 115.00)	101.0 (93.00, 114.00)	0.9751^	105.0 (93.00, 120.00)	105.0 (91.50, 121.00)	102.5 (93.00, 114.00)	0.7523^
Pharmacological DVT prophylaxis use during ICU stay, *n* (%)	746 (91.9)	672 (91.3)	74 (97.4)	0.0655^^	205 (97.6)	137 (97.9)	68 (97.1)	0.7489**
Patient received nephrotoxic drugs/material during ICU stay, *n* (%)*$	731 (90.5)	657 (89.8)	74 (97.4)	0.0314^^	197 (95.2)	129 (94.2)	68 (97.1)	0.3438**
*Comorbidity, n (%)*								
Atrial fibrillation (A Fib)	30 (3.7)	26 (3.5)	4 (5.3)	0.4417**	8 (3.8)	5 (3.6)	3 (4.3)	0.7988**
Left heart failure	60 (7.4)	55 (7.4)	5 (6.6)	0.7837^^	18 (8.6)	14 (10.0)	4 (5.7)	0.2956^^
Right heart failure	60 (9.6)	49 (8.9)	11 (14.5)	0.1253^^	23 (11.0)	16 (11.4)	7 (10.0)	0.7547^^
Pulmonary HTN	122 (19.3)	100 (18.0)	22 (28.9)	0.0231^^	59 (28.1)	41 (29.3)	18 (25.7)	0.5873^^
Hypertension (HTN)	452 (55.5)	411 (55.6)	41 (53.9)	0.7805^^	111 (52.9)	76 (54.3)	35 (50.0)	0.5575^^
Diabetes mellitus (DM)	474 (58.2)	427 (57.8)	47 (61.8)	0.4943^^	114 (54.3)	72 (51.4)	42 (60.0)	0.2398^^
Dyslipidemia (DLP)	179 (22.0)	157 (21.2)	22 (28.9)	0.1225^^	51 (24.3)	30 (21.4)	21 (30.0)	0.1721^^
Ischemic heart disease (IHD)	72 (8.8)	67 (9.1)	5 (6.6)	0.4669^^	12 (5.7)	7 (5.0)	5 (7.1)	0.5283**
Chronic kidney disease (CKD)	53 (6.5)	48 (6.5)	5 (6.6)	0.9775**	12 (5.7)	8 (5.7)	4 (5.7)	> 0.9999**
Cancer	48 (5.9)	45 (6.1)	3 (3.9)	0.4501**	8 (3.8)	5 (3.6)	3 (4.3)	0.7988**
Deep vein thrombosis (DVT)	9 (1.1)	6 (0.8)	3 (3.9)	0.0127**	3 (1.4)	1 (0.7)	2 (2.9)	0.2174**
Pulmonary embolism (PE)	10 (1.2)	10 (1.4)	0 (0.0)	0.3075**	4 (1.9)	4 (2.9)	0 (0.0)	0.1533**
Liver disease (any type)	19 (2.3)	14 (1.9)	5 (6.6)	0.0100**	7 (3.3)	2 (1.4)	5 (7.1)	0.0297**
Stroke	46 (5.6)	41 (5.5)	5 (6.6)	0.7108**	11 (5.2)	7 (5.0)	4 (5.7)	0.8266**

*T Test / ^ Wilcoxon rank sum test is used to calculate the *p*-value

^^Chi square/**Fisher’s Exact teat is used to calculate *p*-value

*$Nephrotoxic medications/ material included IV Vancomycin, Gentamicin, Amikacin, Contrast, Colistin, Furosemide, and/or Sulfamethoxazole/trimethoprim

### Oxygenation parameters

**Fig. 2:**
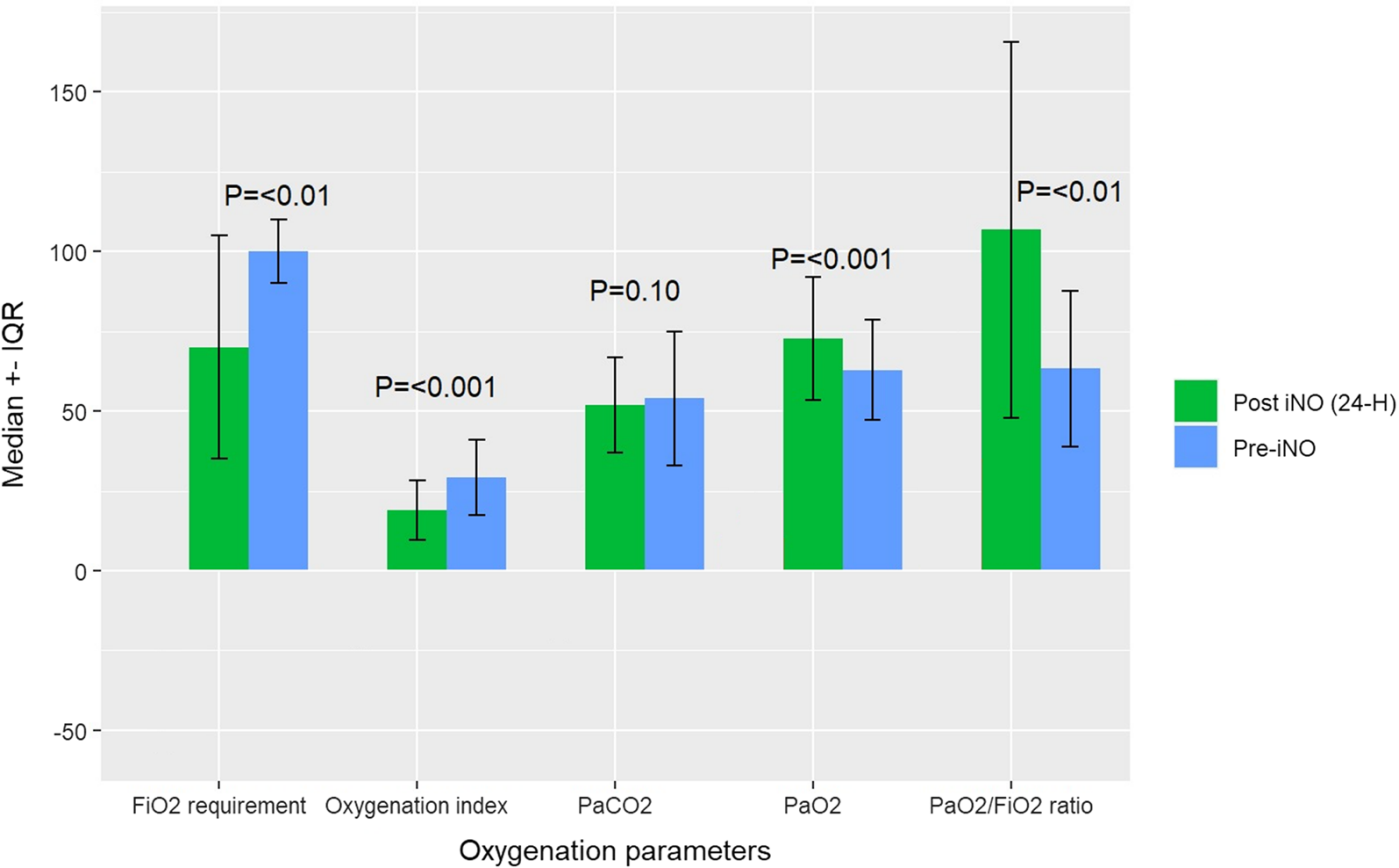
Oxygenation parameters pre-and 24-h post inhaled nitric oxide administration

After 24 h of receiving iNO, the FiO_2_ requirement significantly improved, with a median of 70 (IQR 35); *p* < 0.01. Moreover, the PaO_2_ and PaO_2_/FiO_2_ ratio were significantly improved, with a median of 72.8 (IQR 19.2); *p* < 0.001 and a median of 106.9 (IQR 58.9); *p* < 0.01, respectively. Additionally, the oxygenation index was significantly lower after using iNO, with a median of 18.9 (IQR 9.3); *p* < 0.001. Last, the PaCO2 was lower after receiving iNO; however, it did not reach statistical significance (*p* = 0.10) (Fig. 2).

### Mortality, ventilator-free days, and length of stay

The multivariable Cox proportional hazards regression analyses showed higher 30-day mortality (HR 1.18; 95% CI 0.77, 1.82; *p* = 0.45) and in-hospital mortality in patients who received iNO (HR 1.40; 95% CI 0.94, 2.11; *p* = 0.10) than in patients who did not receive iNO. However, the difference did not reach statistical significance (Table 2).

**Table 2:** The clinical outcomes of critically ill patients with COVID-19 after Propensity score matching

Outcomes	Number of outcomes/Total number of patients			Hazard Ratio (HR) (95%CI)	*p*-value$
	Control	iNO	*p*-value		
30-day mortality, *n* (%)∆	44 (36.1)	41 (73.2)	< 0.0001^^	1.18 (0.77, 1.82)	0.45
In-hospital mortality, *n* (%)∆	52 (41.6)	44 (78.6)	< 0.0001^^	1.40 (0.94, 2.11)	0.10

				Beta coefficient (estimates) (95%CI)	*p*-value $*
Ventilator-free days, mean (SD)	12.0 (11.23)	3.8 (7.38)	< 0.001*	− 1.17 (− 1.79, − 0.54)	< 0.001
ICU length of stay (days), median (Q1,Q3)^∞^	12.0 (8.0, 19.0)	26.0 (19.0, 35.5)	< 0.001^	0.63 (0.32, 0.95)	< 0.001
Hospital length of stay (days), median (Q1,Q3)^∞^	21.0 (13.0, 31.0)	36.0 (28.0, 65.5)	0.002^	0.45 (0.04, 0.87)	0.03

∆Denominator of the percentage is the total number of patients

^**∞**^Denominator is patients who survived

*T -Test / ^Wilcoxon rank sum test is used to calculate the *p*-value

^^Chi-square test is used to calculate the *p*-value

$Cox proportional hazards regression analysis used to calculate HR and *p*-value

$*Generalized linear model is used to calculate estimates and *p*-value

The number of VFDs during the ICU stay at 30 days was significantly lower in the crude and regression analysis in patients who received iNO (beta coefficient (95% CI): − 1.17 (− 1.79, − 0.54), *p* < 0.001). Moreover, both ICU and hospital LOS were significantly longer in patients who received iNO than in the control patients, with beta coefficient (95% CI): 0.63 (0.32, 0.95) and beta coefficient (95% CI): 0.45 (0.04, 0.87), respectively (Table 2).

### Complications during the ICU stay

**Fig. 3:**
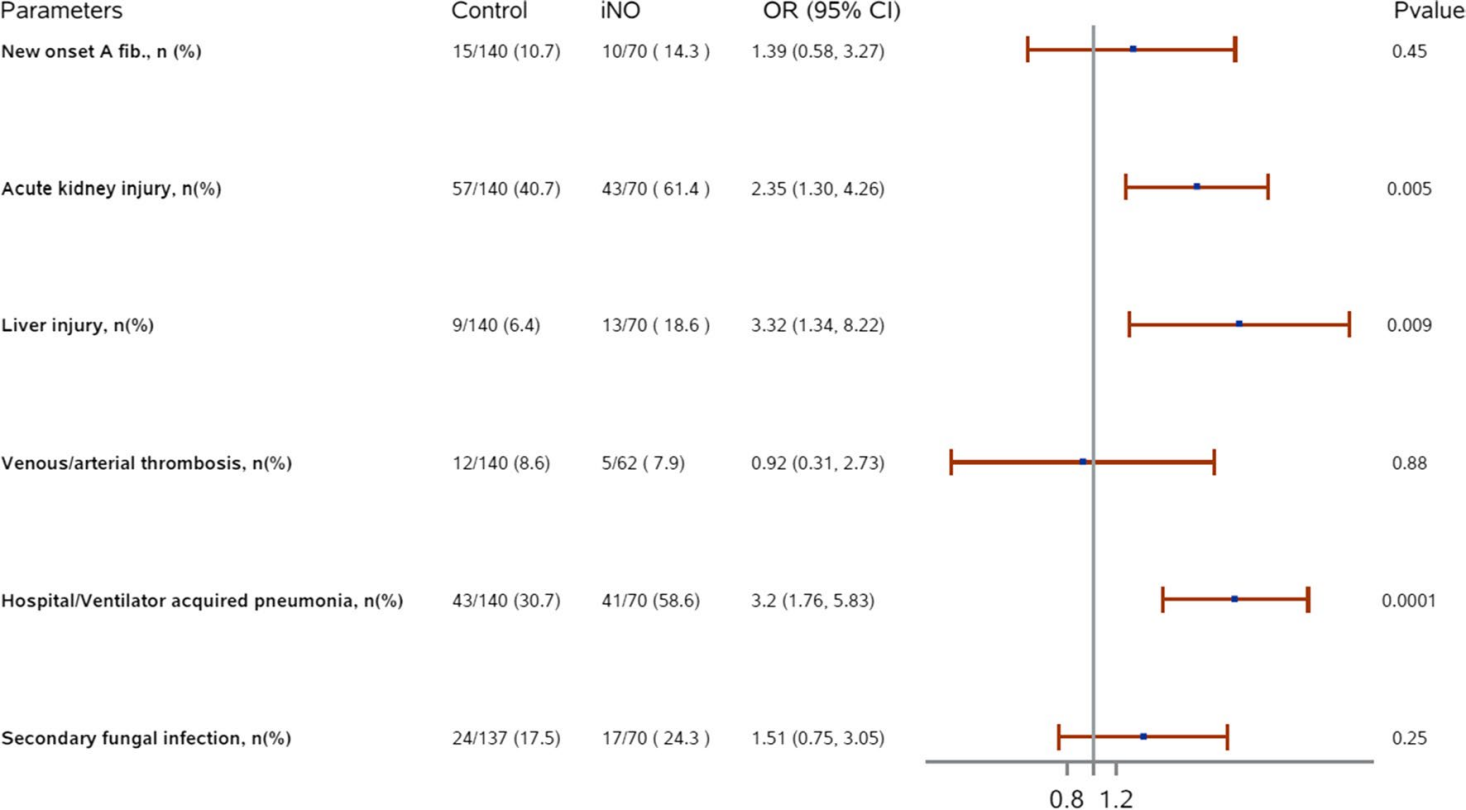
Forest plot of the ICU-acquired complications during stay

Patients who received iNO demonstrated significantly higher odds of AKI (OR (95% CI): 2.35 (1.30, 4.26), *p* = 0.005) (Fig. 3). In addition, the mean delta serum creatinine was significantly increased 24 h post-iNO initiation compared to preiNO in the iNO group (19.6 mmol/l (± 74); *p* value = 0.001). Notably, after PS matching, there was no statistically significant difference in the number of nephrotoxic medications used during the ICU stay between the two groups at baseline (95.2% vs. 97.1%; *p* = 0.34) (Table 1).

The incidence of acute liver injury and pneumonia (HAP/VAP) was significantly higher among the patients who received iNO than control group (OR (95% CI): 3.32 (1.34, 8.22), *p* = 0.009, and OR (95% CI): 3.2 (1.76, 5.83), *p* < 0.001, respectively). At the baseline, patients who received iNO had a significantly higher rate of liver disease (Table 1). On the other hand, there was no statistically significant difference in the incidence of new-onset atrial fibrillation (NOAF), thrombosis events, or secondary fungal infection between the two groups (Fig. 3).

## Discussion

There are limited studies investigating the role of iNO in the management of severe hypoxia due to COVID-19 [32–34]. In this retrospective cohort study, we aimed to assess the improvement in oxygenation parameters 24 h after using a median dose of 40 ppm of rescue iNO for a median duration of 85 h in critically ill COVID-19 patients with moderate to severe ARDS. A significant improvement was observed in our cohort in the PaO_2_, oxygenation index, PaO_2_/FiO_2_ ratio, and FiO_2_ requirement within 24 h after iNO initiation within a median of six days from ICU admission.

Chiara Robba et al., in a prospective observational study conducted to assess the effectiveness of ventilatory rescue strategies in ventilated patients with COVID-19 ARDS, demonstrated that the consumption of iNO improved PaO_2_ (from 65 [67–73] to 72 [67–73] mmHg, *p* = 0.015) and rSO2 (from 53 [51–56] to 57 [55–59] %, *p* = 0.007) [35]. This result was consistent with our findings of the improvement in PaO_2_ with a 10.1% difference post-iNO use.

In patients with COVID-19 and ARDS, significant vascular endothelial damage and a higher incidence of pulmonary microthrombi were previously noted [34]. Most critically ill patients require MV due to difficulty maintaining ventilation and oxygenation, which remains the main challenge in critically ill patients with COVID-19 [34]. In critical care settings, one goal during the COVID-19 pandemic is to increase ventilator-free days to minimize the requirement for respiratory support devices. Thus, enhanced oxygenation caused by smooth muscle vasodilation and increased blood flow to the alveoli is the primary justification for utilizing iNO [36]. In our study, patients with COVID-19 and ARDS who received iNO had significantly fewer VFDs and longer ICU stays. This result contradicts the previously reported finding that receiving iNO did not result in a reduction in the number of VFDs and duration of MV (MD (95% CI)− 0.57 (− 1.82–0.69); I2 = 0%) vs. (MD (95% CI) 1.02 (− 2.08–4.12); I2 = 76%), respectively.

Two observational studies evaluated the use of iNO in patients with COVID-19 and ARDS; both found a lower mortality rate in those patients. In the Parikh et al. study, the mortality rate was 23% of the total patients who received iNO [33]. Ferrari et al. reported that 80% of patients who received 20–30 ppm of iNO survived and were discharged from the hospital [32]. These results are difficult to interpret due to the lack of a comparator group. Our data showed no mortality benefit in patients who received iNO for ARDS secondary to COVID-19. The 30-day and in-hospital mortality hazard ratio were 1.18 (0.77, 1.82) (*p* = 0.45) and 1.40 (0.94, 2.11) (*p* = 0.10), respectively. The timing of initiating iNO therapy and the presence of other risk factors might justify this finding. Acute kidney injury, pneumonia, DM, or infections can influence the mortality rate [37, 38]; it is important to highlight that the SOFA and APACHE II scores were similar between the groups in our study. Our results are in accordance with the results derived from a randomized trial (2004) in ARDS patients, which found that iNO did not improve 28-day mortality [39]. Moreover, Angus et al. reported no difference in survival among the study population at hospital discharge and during the first year. None of the patients included in these studies were patients with COVID-19 and ARDS.

We report a longer ICU stay and hospital LOS in patients who receive iNO (*p* value =  < 0.001). In our study, there was a trend toward an increased risk of AKI, and the rate of hospital/ventilator-acquired pneumonia was significantly higher in the iNO group. These two reasons may have affected the LOS in our study population. Nonetheless, our results agree with a randomized placebo-controlled trial performed by Taylor et al. published in 2004; the researchers restricted their study population to critically ill ARDS patients and found that treatment with iNO did not indicate a reduction in ICU or hospital LOS [39]. Conversely, no studies evaluated iNO effect on the ICU and hospital LOS in COVID-19 patients either with or without ARDS.

Our study demonstrated a possible correlation between the incidence of AKI and the administration of iNO in critically ill patients with COVID-19. This result is consistent with the findings of Wang et al., who identified an increased risk of AKI in ARDS patients with iNO therapy [40]. In our cohort, the occurrence of new-onset atrial fibrillation (NOAF) was not different between the groups. Similar to the results of Koyfman et al., only one patient out of 221 experienced NOAF during intrahospital transport [41]. Pulmonary embolism (PE), DVT, and systemic arterial embolism are common complications in critically ill patients with COVID-19 [42]. Inhaled NO inhibits platelet adhesion and aggregation by reducing fibrinogen binding, which prevents thrombus formation [43]. Our results suggest a possible correlation between the incidence of thrombosis and the administration of iNO. Venous/arterial thrombosis was lower in the iNO group, but the difference was not statistically significant. Notably, most of our patients (97.6%) were on pharmacological DVT prophylaxis.

Nitric oxide (NO) appears to have an immunopathological effect on a host's immunological response and has been shown to decrease type 1 helper T-cell-dependent immune responses. Beyond that, NO and oxygen radicals such as superoxide are essential mediators in the pathophysiology of several infectious illnesses. The biosynthesis of NO is found in a wide range of infectious diseases. Therefore, inducible nitric oxide synthase (NOS) is produced in a large amount over a long time, allowing for the formation of peroxynitrite, a highly reactive nitrogen oxide molecule, which induces oxidative tissue destruction [44]. In addition, patients with COVID-19 who are critically ill are at significant risk of hospital-acquired infections, which are usually caused by MDR bacteria [45]. On the other hand, in a randomized controlled trial, Taylor et al. reported more infection incidents in the low dose iNO group than in the placebo group (66% vs. 41%). Nevertheless, the incidence did not reach statistical significance. This contrasts with our findings, which showed a statistically significant difference in the incidence of ventilator-acquired pneumonia and hospital-acquired pneumonia [39]. Secondary fungal infection did not result in a statistically significant difference. Since most of our patients received steroids and tocilizumab during the first 24 h after admission, we could not completely rule out the effects of such drugs.

To our knowledge, our study is one of the first multicenter studies to investigate the efficacy, safety, ICU stay, and hospital LOS in critically ill patients using iNO for COVID-19–induced moderate-to-severe ARDS. In addition, a propensity matching score was used to decrease the risk of bias and to establish a balance between both groups. However, the limitations of our study include the following. First, this was a retrospective observational study; second, it had a small sample size; third, we did not assess the variety of nitric oxide dosing; last, a short follow-up period may have limited the capturing of other long-term complications. Accordingly, future randomized controlled trials are necessary to support our findings.

## Conclusion

The use of iNO rescue therapy in critically ill COVID-19 patients with moderate-to-severe ARDS is significantly associated with an improvement in oxygenation parameters (PaO_2_, FiO_2_ requirement, P/F ratio, OI) with no mortality benefits. However, iNO use is associated with higher odds of AKI, pneumonia, and LOS, as well as fewer VFDs. Further randomized clinical and interventional studies are required to confirm our findings.

## Supplementary Information

**Additional file 1.** Outcomes definition(s).

## Data Availability

The datasets used and/or analyzed during the current study are available from corresponding author on reasonable request.
