# Supplementary material for: Evaluation of inhaled nitric oxide (iNO) treatment for moderate-to-severe ARDS in critically ill patients with COVID-19: a multicenter cohort study
Source: Crit Care. 2022 Oct 3;26:304. doi: 10.1186/s13054-022-04158-y (PMC9527729; doi:10.1186/s13054-022-04158-y)
Supplement: Supplementary file 1 — Additional file 1. Outcomes definition(s). [file 13054_2022_4158_MOESM1_ESM.docx]

**Outcome definition (s)**

- The 30-day mortality was defined as a death occurring for any cause within 30 days of the admission date during hospital stay; patients who were discharged from the hospital alive were presumed to be survived. All patients were followed until they were discharged from the hospital or died during the in-hospital stay, whichever occurred first.
- Ventilator-free days (VFDs) at 30 days were calculated as the following: if the patients die within 30 days of MV then the VFDs=0, if the patient survived and was successfully liberated from MV then VFDs = 30 − days after MV initiation, and VFDs=0 if the patient is on MV for >30 days.
- Oxygenation index is used to assess severity of hypoxic respiratory failure. Oxygenation index = (FiO2 x PAW) / PaO2; where variables are as follows: FiO2 = fraction of inhaled oxygen, %, PAW = mean airway pressure, mm Hg and PaO2 = Partial pressure of arterial oxygen, mm Hg. A lower oxygenation index is better.
- Acute kidney injury (AKI) was defined as a sudden decrease of renal function within 48 hours, defined by an increase in absolute SCr of at least 26.5 μmol/L (0.3 mg/dL) or by a percentage increase in SCr ≥ 50% (1.5× baseline value) during ICU stay._._
- Acute liver injury was defined as alanine aminotransferase (ALT) exceeding three times the upper limit of normal or double in patients with elevated baseline ALT during the ICU stay.(23)
- Secondary fungal infection was identified through the blood, urine, wound, drainage, cerebrospinal fluid, and/or respiratory cultures. Cultures were excluded if the laboratory reported them as a "contaminant sample."The fungal growth was considered significant if the growth was ≥ 100,000 colony forming units (CFUs)/ml in sputum or endotracheal aspiration, ≥10,000 CFUs of single organism/ml in bronchoalveolar lavage or ≥ 1000 CFUs of single organism/ml in protected specimen brushes. Additionally, urinary cultures were considered significant if showing a growth ≥100,000 CFUs/ml of no more than two species of microorganisms.(24)
- Respiratory failure was defined as either low arterial carbon dioxide tension (PaCO_2_) or hypoxemic respiratory failure (PaO_2_ < 60 mm Hg with a normal or hypercapnic respiratory failure (PaCO_2_ > 50 mm Hg) that requires mechanical ventilation.(25)
- Inhaled nitric oxide responder defined as 20% increase or more of arterial PaO2 within 24 hours of iNO initiation.
